# Supplementary material for: Pitch Gradation by Ion-Dragging Effect in Polymer-Stabilized Cholesteric Liquid Crystal Reflector Device
Source: Polymers (Basel). 2020 Jan 4;12(1):96. doi: 10.3390/polym12010096 (PMC7023619; doi:10.3390/polym12010096)
Supplement: Supplementary file 1 [file polymers-12-00096-s001.pdf]

Support Information for

Pitch Gradation by Ion-Dragging Effect in Polymer Stabilized Cholesteric Liquid Crystal Reflector Device

Xiaowen Hu [1,2], Weijie Zeng [1,2], Qiumei Nie [1,2], Xinmin Zhang [1,2], Kai Wang [3], Xiaoling Liao [1,2], Xinshuai Jiang [2], Xiao-Fang Jiang [2,*], Mingliang Jin [2], Lingling Shui [2] and Guofu Zhou[1,2,4]

1 SCNU-TUE Joint Lab of Device Integrated Responsive Materials (DIRM), National Center for International Research on Green Optoelectronics, South China Normal University, Guangzhou 510006, P. R. China.
2 Guangdong Provincial Key Laboratory of Optical Information Materials and Technology & Institute of Electronic Paper Displays, South China Academy of Advanced Optoelectronics, South China Normal University, Guangzhou 510006, P. R. China.
3 Department of Materials Science and Engineering, Pennsylvania State University, University Park, PA 16802, United States.
4 Shenzhen Guohua Optoelectronics Tech. Co. Ltd., Shenzhen 518110, China
* Corresp*onde*nce: jiangxf@scnu.edu.cn (X-F. J.);

Content

Supplementary Figures 2

Supplementary Figures

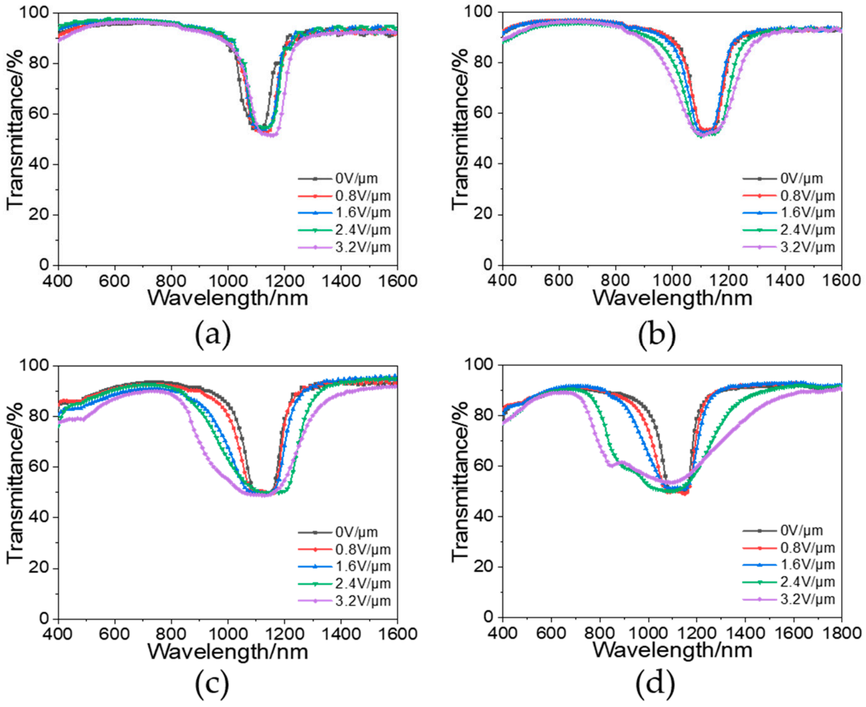

Figure S1. The reflection bandwidth of a cell with different thicknesses versus different in situ DC bias treatments (a)5μm, (b)15μm, (c)25μm and (d)40μm.
